# Preprocessing, normalization and integration of the Illumina HumanMethylationEPIC array with minfi

**DOI:** 10.1093/bioinformatics/btw691

**Published:** 2016-11-29

**Authors:** Jean-Philippe Fortin, Timothy J. Triche, Kasper D Hansen

**Affiliations:** 1 Department of Biostatistics, Johns Hopkins Bloomberg School of Public Health, Baltimore, MD, USA; 2 Jane Anne Nohl Division of Hematology, Keck School of Medicine of USC, Los Angeles, CA, USA; 3 McKusick-Nathans Institute of Genetic Medicine, Johns Hopkins School of Medicine, Baltimore, MD, USA

## Abstract

**Summary:**

The *minfi* package is widely used for analyzing Illumina DNA methylation array data. Here we describe modifications to the *minfi* package required to support the HumanMethylationEPIC (‘EPIC’) array from Illumina. We discuss methods for the joint analysis and normalization of data from the HumanMethylation450 (‘450k’) and EPIC platforms. We introduce the single-sample Noob (*ssNoob*) method, a normalization procedure suitable for incremental preprocessing of individual methylation arrays and conclude that this method should be used when integrating data from multiple generations of Infinium methylation arrays. We show how to use reference 450k datasets to estimate cell type composition of samples on EPIC arrays. The cumulative effect of these updates is to ensure that *minfi* provides the tools to best integrate existing and forthcoming Illumina methylation array data.

**Availability and Implementation:**

The minfi package version 1.19.12 or higher is available for all platforms from the Bioconductor project.

**Supplementary information:**

Supplementary data are available at *Bioinformatics* online.

## 1 Introduction

The IlluminaHumanMethylation450 (‘450k’) array is a widely used platform for assaying DNA methylation in a large number of samples (Bibikova *et al.*, 2011), and has been the platform of choice for epigenome-wide association studies and large-scale cancer projects. In 2015, Illumina released their next generation methylation array, the HumanMethylationEPIC (‘EPIC’) array (Moran *et al.*, 2016), with almost twice the number of CpG loci. This increased resolution, coupled with greatly expanded coverage of regulatory elements, makes the EPIC array an attractive platform for large-scale profiling of DNA methylation.

The minfi package in R/Bioconductor (Gentleman *et al.*, 2004; Huber *et al.*, 2015) is a widely used software package for analyzing data from the Illumina HumanMethylation450 array (Aryee *et al.*, 2014). In addition to the analysis methods provided in the package, it exposes a flexible framework for handling DNA methylation data.

## 2 Methods and results

We have extended the minfi package to support EPIC arrays. This includes functionality to (i) convert an EPIC array to a virtual 450k array for joint normalization and processing of data from both platforms, (ii) estimate cell type proportions for EPIC samples using external reference data from the 450k array. In addition, we present a new single-sample normalization method (ssNoob) for methylation arrays. Concurrently, we have extended the shinyMethyl package (Fortin *et al.*, 2014b) for interactive QC of Illumina methylation arrays.

Following the release of the EPIC chip, Illumina quickly released multiple versions of the manifest file describing the array design, as well as DMAP files used by the scanner. As a consequence, multiple types of IDAT files containing the raw data can be encountered in the wild. Addressing this has required more robust parsing code in minfi. It is therefore highly recommended that users analyzing EPIC arrays aggressively keep minfi and associated annotation packages updated.

A substantial percentage (93.3%) of loci contained on the 450k array are also present on the EPIC array, measured using the same probes and chemistry. That makes it possible to combine data from both arrays. The lowest level of the combination can occur at the probe level. We have implemented this functionality in the function combineArrays which outputs an object that behaves either as a 450k or an EPIC array as chosen by the user with a reduced number of probes; we call this is a virtual array. We also support the combination of the two array types at the CpG locus level after the creation of the methylation and unmethylation channels.

### 2.1 Single sample normalization with ssNoob

Single sample normalization is of great potential benefit to users, particularly for analyzing large datasets which arrive in batches, because data can be processed separately and independently of the previously processed data. We adapted the Noob method (Triche *et al.*, 2013) to be a single sample normalization method by removing the need for a reference sample in the dye bias equalization procedure step. We call the method ‘ssNoob’, and details of the algorithm are provided in the Supplementary Methods. We note that on the Beta value scale, there is no difference between values returned by Noob or ssNoob (Supplementary Methods). Differences are confined to the methylated and unmethylated signals.

**Fig. 1.**
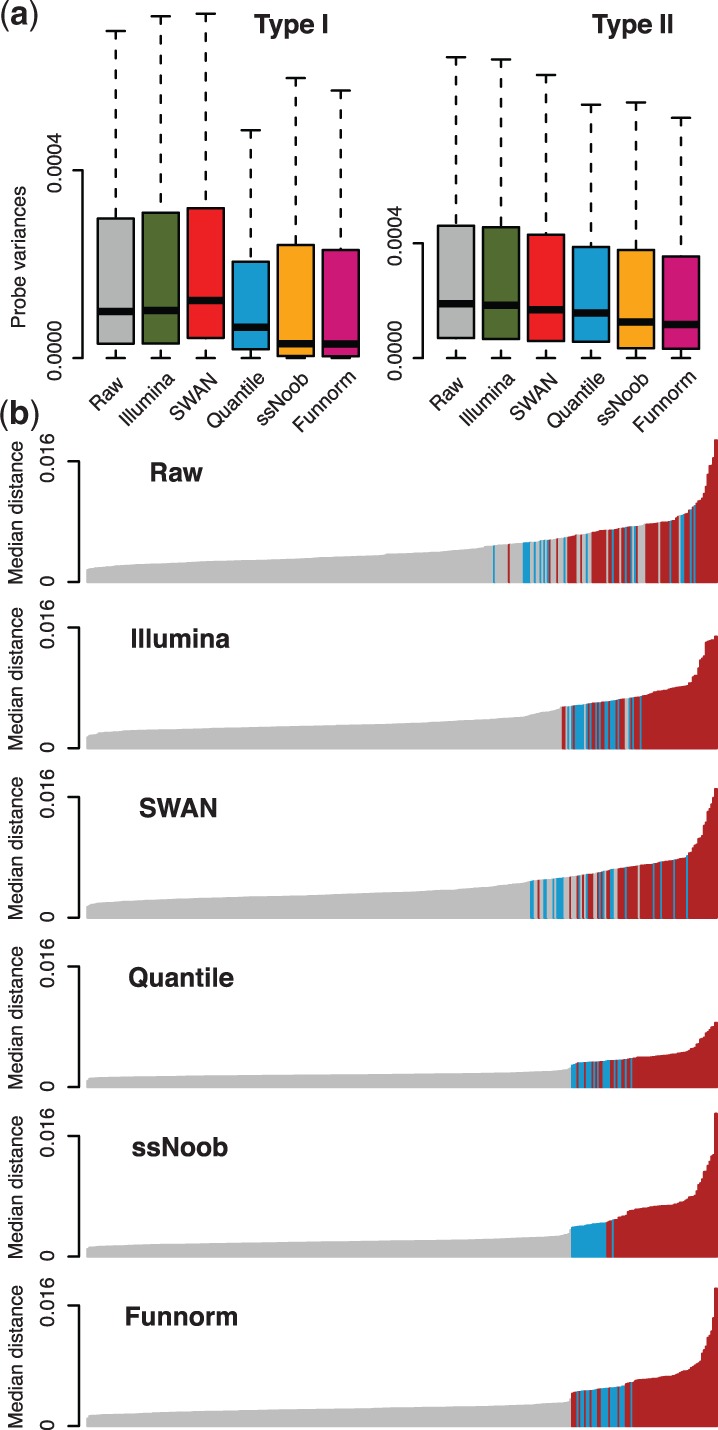
(**a**) Distribution of the variance between technical replicates assayed on the EPIC array, preprocessed using various methods. (**b**) The median distance between LCLs measured on the EPIC array and a number of different samples (261 LCLs in grey, 20 PBMC in blue and 58 ENCODE cell lines in red). All samples (both EPIC and 450k) were combined into a virtual array prior to normalization

**ssNoob reduces technical variation.** We assessed how the different preprocessing methods perform at reducing technical variation among three technical replicates of the cell line GM12878 assayed on the EPIC array: preprocessing as Illumina, SWAN normalization (Maksimovic *et al.*, 2012), stratified quantile normalization (Aryee *et al.*, 2014), ssNoob (Triche *et al.*, 2013), functional normalization (Fortin *et al.*, 2014a) and no normalization. We calculated the variance of the Beta values across the three technical replicates at each CpG, stratified by probe design type. Boxplots of the distribution of these variances are shown in Figure 1a. The results show that relative performance of the different preprocessing methods is similar on the EPIC array to what we previously observed on the 450k array; we caution that we also previously found that reduction in technical variation is not always associated with improvements in replication between studies (Fortin *et al.*, 2014a).

**ssNoob improves classification across array types.** We assessed the performance of the above normalization methods when 450k and EPIC data are first combined at the probe level, and then subsequently normalized together. We compared the three EPIC technical replicates to a set of 450k arrays collated from publicly available data (Supplementary Table S1). This set consists of 261 lymphoblastoid cell lines (LCLs), the same cell type as GM12878, along with 20 peripheral blood mononuclear (PBMC) samples and 58 other samples from ENCODE.

We computed the median distance between data from the EPIC array and all of the 450k data after normalization. A useful normalization strategy will result in the LCLs drawing closer to each other while moving further from the other cell types. We used the distance as a metric for predicting whether or not a 450k sample is an LCL sample, and displayed prediction performance as a ROC curve (Supplementary Fig. S1). While all methods predict well, we observe that ssNoob, functional normalization and quantile normalization achieved perfect prediction performance. We then investigated whether or not the methods can separate the PBMC samples from the ENCODE samples (Fig. 1b, Supplementary Fig. S3), and observe that here ssNoob performed best, followed by functional normalization and quantile normalization.

We repeated the same assessments when normalizing EPIC samples separately from 450k samples, then combining the data after normalization (Supplementary Figs S1–S3). Here quantile normalization performed worse, as expected. As ssNoob is a single-sample procedure, it is not affected by whether samples are combined or not prior to normalization.

Based on this assessment, and on the performance of Noob in existing benchmarks, we conclude that ssNoob is the best performing method for joint normalization of data from the EPIC and 450k arrays. We caution that this evaluation is based on a small number of EPIC samples and should therefore be considered preliminary.

### 2.2 Estimating cell-type composition for EPIC arrays using 450k reference data

Several methods have been proposed to estimate the cell-type proportions from reference datasets made of sorted samples (Houseman *et al.*, 2012; Jaffe and Irizarry, 2014), and several reference datasets exist for the 450k array (Bakulski *et al.*, 2016; Guintivano *et al.*, 2013; Reinius *et al.*, 2012). We adapted the function estimateCellCounts to estimate cell type proportions of EPIC samples using 450k reference datasets. Briefly, the EPIC dataset is converted into a virtual 450k dataset and cell type proportions are estimated using probes common to both arrays. To evaluate how removing 7% of probes from the 450k platform impacts the cell-type composition estimation for EPIC arrays, we estimated whole-blood cell-type proportions for the 20 PBMC samples, before and after removing the probes that differ between the 450k and EPIC arrays. This yielded very good results; for each cell type, the correlation of the cell type proportions between the two sets of data is higher than 0.99 (Supplemental Fig. S4). As noted, reference datasets are also available for cord blood and brain.

### 2.3 Summary of the functionality in minfi

Most functionality in minfi supports all generations of Illumina Infinium HumanMethylation arrays: 27k, 450k and EPIC. This includes the different preprocessing & normalization functions, as well as differential analysis tools: dmpFinder for differentially methylated positions (DMPs), bumphunter for differentially methylated regions (DMRs) and blockFinder for differentially methylated blocks (DMBs). We have also adapted the recent function compartments (Fortin and Hansen, 2015), which estimates A/B compartments as revealed by Hi-C data, to the EPIC array. The main functions in minfi are presented as Table 1.

**Table 1.** Main functions in the minfi package

Function	Description	Platforms
**Data acquisition**		
read.metharray	Read idat files into R	27k, 450k, EPIC
convertArray	Cast an array platform into another	27k, 450k, EPIC
combineArrays	Combine data from different platforms	27k, 450k, EPIC
**Quality control**		
getSex	Estimation of the samples sex	27k, 450k, EPIC
getQC	Estimation of sample-specific QC	27k, 450k, EPIC
qcReport	Produces a PDF QC report	27k, 450k, EPIC
**Preprocessing**		
preprocessRaw	No normalization	27k, 450k, EPIC
preprocessQuantile	(Stratified) quantile normalization	27k, 450k, EPIC
preprocessIllumina	Genome Studio normalization	27k, 450k, EPIC
preprocessSWAN	SWAN normalization	450k, EPIC
preprocessNoob	Background and dye bias correction	27k, 450k, EPIC
preprocessFunnorm	Functional normalization	450k, EPIC
**Differential analysis**		
dmpFinder	Estimation of DMPs	27k, 450k, EPIC
bumphunter	Estimation of DMRs	27k, 450k, EPIC
blockFinder	Estimation of DMBs	450k, EPIC
**Other useful functions**		
compartments	Estimation of A/B compartments	450k, EPIC
estimateCellCounts	Estimation of cell-type proportions	27k, 450k, EPIC
addSnpInfo	Intersect probes with dbSNP	27k, 450k, EPIC

## Supplementary Material

Supplementary Data
