# Supplementary material for: Preprocessing, normalization and integration of the Illumina HumanMethylationEPIC array with minfi
Source: Bioinformatics. 2016 Nov 29;33(4):558–60. doi: 10.1093/bioinformatics/btw691 (PMC5408810; doi:10.1093/bioinformatics/btw691)
Supplement: Supplementary Data [file btw691_supp.pdf]

Supplementary Material
for
Preprocessing, normalization and integration of the Illumina HumanMethylationEPIC array with minfi

Jean-Philippe Fortin[1], Timothy J. Triche, Jr.[2], and Kasper D. Hansen[1,3,*]

[1]Department of Biostatistics, Johns Hopkins Bloomberg School of Public Health
[2]Jane Anne Nohl Division of Hematology, Keck School of Medicine of USC
[3]McKusick-Nathans Institute of Genetic Medicine, Johns Hopkins School of Medicine

*To whom correspondence should be addressed. Email: khansen@jhsph.edu

Supplementary Methods

Data

Supplementary Table 1. Methylation datasets.

Dataset	Cell type	n	Type Replicates	Platform	Accession	Reference
EPIC-LCL	LCL (GM12878)	3	Technical	EPIC	-	Illumina website, also available in minfiDataEPIC (Fortin and Hansen, 2016)
450k-Esteller	PBMC	20	Biological	450k	GSE36369	Heyn et al. (2013)
	LCL	256	Biological	450k	GSE36369	Heyn et al. (2013)
450k-ENCODE	LCL (GM12878)	1	-	450k	GSE40699	ENCODE Project Consortium (2004)
	LCL (Others)	4	Biological	450k	GSE40699	ENCODE Project Consortium (2004)
	Others	58	Biological	450k	GSE40699	ENCODE Project Consortium (2004)

Single sample normalization with ssNoob

For single sample preprocessing, we eliminated the use of a reference sample r in correcting the dye bias ratio R_{UM} estimated from normalization controls (c_{AT} for U, c_{GC} for M). Previously (Triche et al., 2013), we computed the corrected $\widetilde{\beta}_{ij}$ at probe j in sample i as

$$\widetilde{\beta}_{ij} = \frac{\widetilde{M}_{ij}}{\widetilde{M}_{ij} + \widetilde{U}_{ij}}$$

where the corrected methylated and unmethylated intensities are estimated as

$$\widetilde{M}_{ij} = \frac{M_{ij} I_{\text{ref}}}{\widehat{c_{GC_i}}}, \qquad \widetilde{U}_{ij} = \frac{U_{ij} I_{\text{ref}}}{\widehat{c_{AT_i}}}$$

with

$$I_{\text{ref}} = \frac{\widehat{c_{GC_r}} + \widehat{c_{AT_r}}}{2}, \qquad r = \arg\min_r \left| \frac{\widehat{c_{GC_r}}}{\widehat{c_{AT_r}}} - 1 \right|.$$

Here the methylated M_{ij} and unmethylated intensities U_{ij} are background corrected as described in Triche et al. (2013), and $\widehat{c_{GC_i}}, \widehat{c_{AT_i}}$ are the averages of the normalization controls on array i. We note that background correction is a single sample procedure.

Note that, since the reference normalization control intensity I_{ref} vanishes when we convert the corrected $\widetilde{M}_{ij}$ and $\widetilde{U}_{ij}$ into $\widetilde{\beta}_{ij}$, it has no impact on the corrected Beta values. We address the dye bias in Type II probes by correcting U_{ij} as

$$\widetilde{U}_{ij} = \frac{U_{ij}}{R_{UM_i}}, \qquad R_{UM_i} = \frac{\widehat{c_{AT_i}}}{\widehat{c_{GC_i}}}$$

recovering the dye bias corrected Beta values as

$$\beta_{ij} = \frac{M_{ij}}{M_{ij} + \widetilde{U}_{ij}}.$$

The resulting β_{ij} is identical to reference-based $\widetilde{\beta}_{ij}$, and accuracy of detection p-values (based on intensities) may also benefit from the reduced manipulation of raw intensities.

Normalization Assessment

Normalization on separate arrays. For each normalization method, we normalized the EPIC and 450k data separately. We then combined the two normalized array datasets at the CpG level, resulting in a matrix of Beta values $\mathbf{B}^{\text{Sep}}$ with 453,093 rows and 342 columns (loci only measured on the EPIC was discarded for our assessment).

Normalization on virtual array. We combined the unnormalized EPIC and 450k data at the probe level to create a virtual array. We then normalized the data jointly by applying each normalization method to the combined virtual array, resulting in a matrix of Beta values $\mathbf{B}^{\text{Comb}}$ with 453,093 rows and 342 columns.

Median distance of the 450k samples relative to the EPIC array. For both $\mathbf{B}^{\text{Sep}}$ and $\mathbf{B}^{\text{Comb}}$, we compute the average methylation Beta value profile of the GM12878 cell line assayed on the EPIC array by taking the mean of the three technical replicates, resulting in the two vectors of Beta values $B_{\text{EPIC}}^{\text{Sep}}$ and $B_{\text{EPIC}}^{\text{Comb}}$. For each normalized 450k sample B_i^{Sep} and B_i^{Comb}, we calculate the median distances m_i^{Sep} and m_i^{Comb} with respect to the EPIC array as follows:

$$m_i^{\text{Sep}} = \text{median}[(B_{\text{EPIC}}^{\text{Sep}} - B_i^{\text{Sep}})^2]$$
$$m_i^{\text{Comb}} = \text{median}[(B_{\text{EPIC}}^{\text{Comb}} - B_i^{\text{Comb}})^2].$$

The median distance values m_i^{Sep} and m_i^{Comb} quantify the similarity of each 450k sample with respect to the cell line GM12878 sample assayed on the EPIC array. We present the ordered values m_i^{Sep} and m_i^{Comb} in Figures S2 and **??** respectively. These barplots are colored by tissue type.

For each normalization method, we use the median distances as predictor of cell type (LCL or not LCL) for the 450k samples, and use an ROC curve to summarize the specificity and sensitivity of each normalization method (Figure S1).

Software

The results in this manuscript were produced using minfi version 1.19.12, minfiDataEPIC version 0.99.3, IlluminaHumanMethylationEPICmanifest version 0.3.0, and IlluminaHu-

manMethylationEPICanno.ilm10b2.hg19 version 0.3.0. Scripts describing our reproducible analysis are available at https://github.com/hansenlab/EPIC450k_repro.

Bibliography

ENCODE Project Consortium (2004). "The ENCODE (ENCyclopedia Of DNA Elements) Project". *Science* 306.5696, pp. 636–640. DOI: 10.1126/science.1105136.

Fortin, J.-P. and K. D. Hansen (2016). *minfiDataEPIC: Example data for the Illumina Methylation EPIC array*. R package version 0.99.3. URL: http://www.bioconductor.org/packages/minfiDataEPIC.

Heyn, H., S. Moran, I. Hernando-Herraez, S. Sayols, A. Gomez, J. Sandoval, D. Monk, K. Hata, T. Marques-Bonet, L. Wang, and M. Esteller (2013). "DNA methylation contributes to natural human variation." *Genome Research* 23.9, pp. 1363–1372. DOI: 10.1101/gr.154187.112.

Triche, T. J., D. J. Weisenberger, D. Van Den Berg, P. W. Laird, and K. D. Siegmund (2013). "Low-level processing of Illumina Infinium DNA Methylation BeadArrays". *Nucleic Acids Research* 41.7, e90. DOI: 10.1093/nar/gkt090.

Supplemental Figures

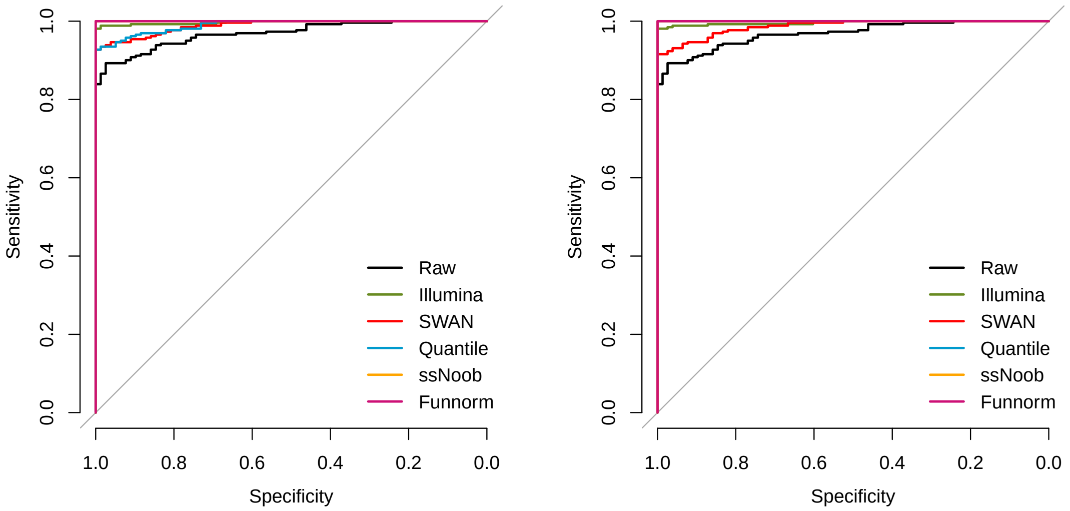

(a) Separate normalization **(b)** Combined normalization

Supplemental Figure S1. Normalization assessment using ROC curves. The median distance between LCLs measured on the EPIC array and a number of different samples measured using the 450k array was used to predict whether a 450k sample was a LCL or not. Displayed is an ROC curve showing the performance of the predictor. (a) EPIC and 450k samples were combined into a virtual array and then subsequently normalized together. (b) EPIC and 450k samples were normalized separately and then subsequently combined at the methylation loci level.

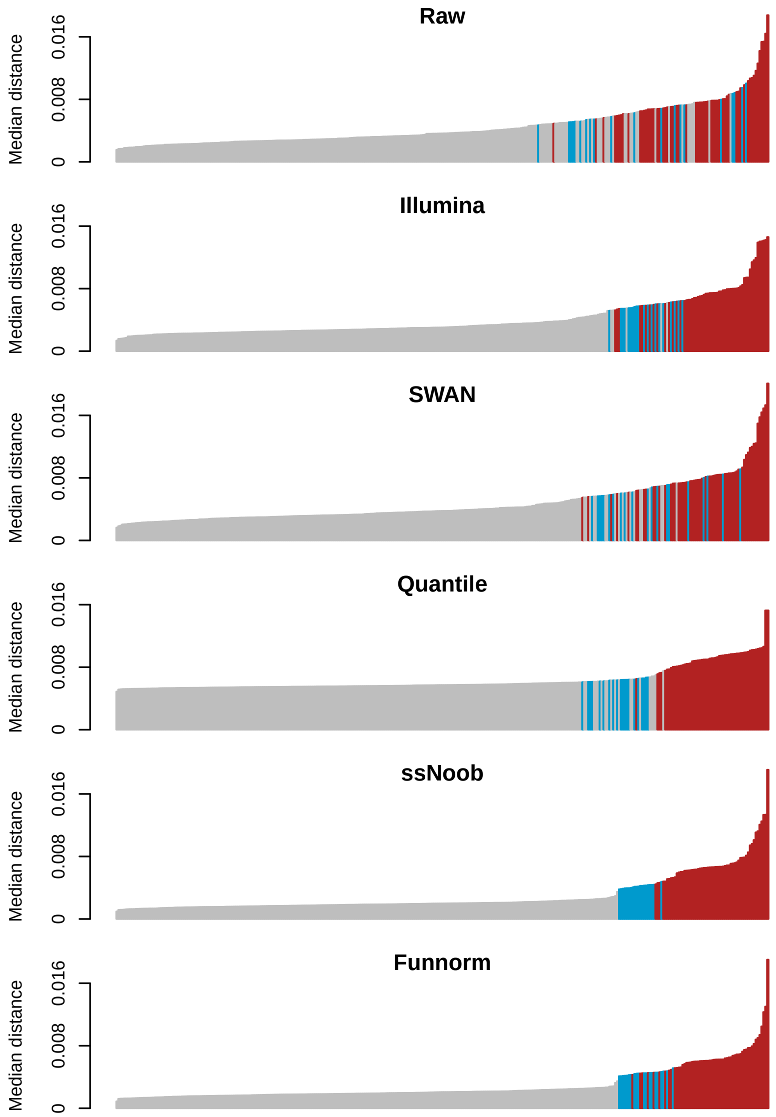

Supplemental Figure S2. Median distance between 450k samples and the average EPIC array sample for normalization on separate arrays. As Figure 1b, but EPIC and 450k data were normalized separately and subsequently combined.

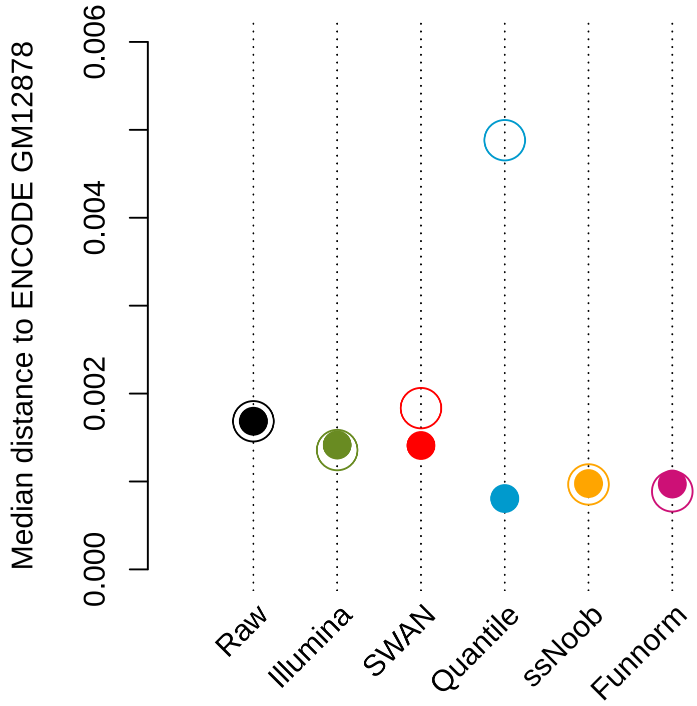

Supplemental Figure S3. Median distance between the 450k ENCODE GM12878 sample and the average EPIC array sample for different normalizations. The full dots represent the median distances for data normalized after the creation of a virtual array, while the circles represent the median distances when the EPIC and 450k data are normalized separately and subsequently combined.

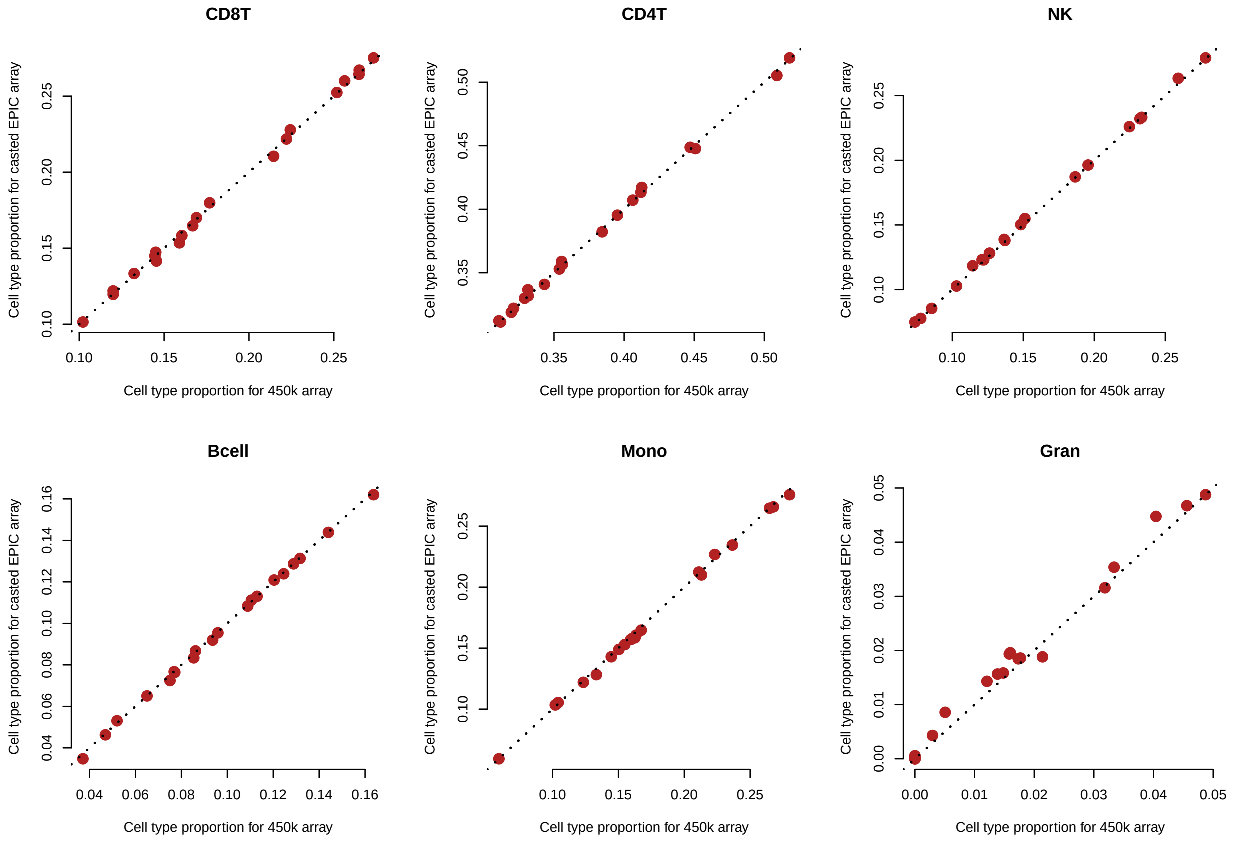

Supplemental Figure S4. Cell-type composition proportion estimates for the 20 PBMC samples For the 6 different cell types, we plot on the x-axis the cell-type proportions estimated using all of the 450k probes, and we plot on the y-axis the cell-type proportions estimated using probes that are common between the 450k array and the EPIC array.
